# Omega‐3 fatty acids are associated with blood–brain barrier integrity in a healthy aging population

**DOI:** 10.1002/brb3.2273

**Published:** 2021-07-29

**Authors:** Samuel Barnes, Shilpy Chowdhury, Nicole M. Gatto, Gary E. Fraser, Grace J. Lee

**Affiliations:** ^1^ Department of Radiology Loma Linda University Medical Center Loma Linda California USA; ^2^ Department of Nutrition School of Public Health Loma Linda University Loma Linda California USA; ^3^ School of Community and Global Health Claremont Graduate University Claremont California USA; ^4^ Center for Nutrition Healthy Lifestyle and Disease Prevention School of Public Health Loma Linda University Loma Linda California USA; ^5^ Department of Psychology School of Behavioral Health Loma Linda University Loma Linda California USA

**Keywords:** blood–brain barrier, MRI, n‐3 PUFA, neurocognitive tests

## Abstract

In aging populations, omega‐3 polyunsaturated fatty acids (PUFAs) have been associated with better cognitive function, slower rates of cognitive decline, and lower risk of developing dementia. Animal studies have shown that diets rich in omega‐3 PUFAs reduce blood–brain barrier (BBB) disruption associated with aging, but this has yet to be observed in humans.

Forty‐five healthy subjects (mean age, 76 years) were recruited and underwent cognitive assessment (verbal learning and memory, language, processing speed, executive function, and motor control) and measurement of PUFAs. Forty of the same subjects also underwent magnetic resonance imaging (MRI) to measure BBB integrity (*K*_trans_ using dynamic contrast‐enhanced MRI).

The long chain omega‐3 score (DHA+EPA) was negatively correlated with *K*_trans_ values in the internal capsule, indicating higher omega‐3 levels were associated with greater BBB integrity in this region (*r* = –0.525, *p* = .004). Trends were observed for a positive correlation between the long chain omega‐3 score and both memory and language scores, but not with executive function, speed, or motor control. The omega‐6 score was not significantly correlated with any cognitive scores or *K*_trans_ values.

The significant correlations between long chain omega‐3 levels and BBB integrity provide a possible mechanism by which omega‐3 PUFAs are associated with brain health.

## Graphical abstract

This work measured cognition, blood‐brain barrier integrity, and long chain omega‐3 levels in 45 health older adults. We found significant correlations between long chain omega‐3 levels and BBB integrity, providing evidence of a possible mechanism by which omega‐3 may contribute to brain health.

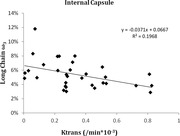

## 1 INTRODUCTION

As no cure for Alzheimer's disease (AD) has yet been discovered and treatment options are limited, there is an urgent need to focus efforts on primary prevention as the number of adults with the disease is expected to triple over the next three decades (Brookmeyer et al., 2007). Identification of modifiable risk factors such as diet and understanding their mechanisms of action are critical to interventions. Omega‐3 polyunsaturated fatty acids (PUFAs), which are predominantly received through dietary sources, have been associated with better cognitive function (Bowman et al., 2012; Kalmijn et al., 2004; van Gelder et al., 2007), slower rates of cognitive decline (van Gelder et al., 2007), and an overall lower risk of developing dementia (Barberger‐Gateau et al., 2007; Morris et al., 2003). Neuroanatomically, higher omega‐3 PUFA intake has been associated cross‐sectionally with larger gray matter volume in healthy older adults (Conklin et al., 2007; Tan et al., 2012; Titova et al., 2013) and longitudinally with lower rates of atrophy in the hippocampus and amygdala in community‐dwelling adults over the age of 65 (Samieri et al., 2012). However, the basis for these relationships is not completely understood in humans.

Research on animals has been highly suggestive that diet impacts cognition through maintenance of the blood–brain barrier (BBB) (Davidson et al., 2012; Kanoski et al., 2010; Sparks et al., 2000; Takechi et al., 2013). Animal models have shown that diets rich in omega‐3 PUFAs reduce BBB disruption associated with aging (Kuo et al., 2010) and poor diet (Takechi et al., 2013). The BBB has long been implicated in neurodegeneration and AD (Farrall & Wardlaw, 2009; Zlokovic, 2011 ), and omega‐3 PUFA supplementation in murine models decreased the amount of Aβ and neuronal loss in the brain (Hooijmans et al., 2012).

Existing studies of the BBB in humans (Farrall & Wardlaw, 2009) have almost entirely relied on either examinations of the cerebral spinal fluid (CSF), which gives no spatial information about where leakage occurs and requires an invasive lumbar puncture, or positron emission tomography (PET) and computed tomography (CT) exams, which utilize harmful radiation. Previous magnetic resonance imaging (MRI) studies of the BBB have had reduced sensitivity due to limited data acquisition and postprocessing techniques (Starr et al., 2009; Su et al., 1998; Wang et al., 2006). More sophisticated dynamic contrast enhanced (DCE) MR techniques have recently been developed, which allow accurate detection of subtle changes in BBB integrity (Barnes et al., 2015; Cramer et al., 2015; Montagne et al., 2015; Taheri et al., 2011). These new advances allow cognitive decline and BBB integrity to be carefully and noninvasively examined in humans. They have also provided evidence to suggest that reduced BBB integrity is a key mechanism early in cognitive decline and AD (Huisa et al., 2015; Montagne et al., 2015; Nation et al., 2019). Specifically, studies have shown a reduction of BBB integrity in the hippocampus to be associated with aging and additional loss of BBB integrity in individuals with mild cognitive impairment, a stage preceding dementia, compared to cognitively intact age‐matched controls (Montagne et al., 2015). However, whether specific dietary factors, including omega‐3 PUFAs, are associated with preserving BBB integrity has not yet been investigated in humans.

This study explores the relationship among omega‐3 PUFAs, cognitive function, and BBB integrity in a healthy elderly adult population, thereby secondarily considering the utility of measuring BBB integrity with MRI as an early, noninvasive, and sensitive biomarker of cognitive impairment risk. We hypothesize that higher levels of omega‐3 PUFAs will be inversely associated with *K*_trans_ levels measured using DCE MRI, an indicator of BBB integrity, which may help to preserve cognitive function in aging. We preliminarily investigate whether associations are specific to brain regions for which BBB changes could affect cognition.

## 2 METHODS

### 2.1 Participants

The Adventist Health Study‐2 (AHS‐2) is a prospective cohort study of over 96,000 members of the Seventh Day Adventist Church in the United States and Canada. During 2002–2007, mostly Caucasian (65.3%) and African‐American (26.9%) adult men and women with a mean age of 59 years (range 30–110 years) were enrolled and completed a 50‐page baseline questionnaire, which included sections on dietary, demographic, anthropometric, and lifestyle factors (Butler et al., 2008). The cohort is healthy: at baseline, high proportions reported being in excellent health. Forty‐five percent of cohort members follow vegetarian diets (Rizzo et al., 2013) and nonvegetarians consume lower amounts of meat compared to the general US population (Rizzo et al., 2013; Tantamango‐Bartley et al., 2013). Note that 1.1% are current smokers and 6.6% drink alcohol (Butler et al., 2008). About 28% had omega‐3 scores >8% (considered low vascular risk, Harris & Von Schacky, 2004), 65% had scores from 4% to 8% (intermediate vascular risk), and 7% had scores ≤4% (high vascular risk). Since the cohort has aged, and the majority of the cohort is elderly, the AHS‐2 presents an opportunity to study age‐related chronic diseases.

In 2016, we identified 2685 members of the cohort for whom study records indicated were 60 years or older, community‐dwelling and living within 75 miles of Loma Linda University (LLU). During 2016–2018, 199 were reached by telephone and invited to participate in the AHS‐2 Cognitive and Neuroimaging (AHS2‐CAN) substudy (Gatto et al., 2020). Of those, 168 (84%) agreed to participate and were screened for eligibility. Participants were excluded if they did not understand and speak English proficiently or had any acute medical conditions that could adversely impact cognitive function. One hundred and thirty‐two otherwise healthy adults were enrolled in the AHS2‐CAN study and completed the baseline study procedures, which included cognitive and physical assessments. Approximately 1 year later, participants were invited to return for a follow‐up cognitive assessment and brain MRI scan. Additional exclusions were made for a diagnosed neurological condition, such as Parkinson's disease, epilepsy, or multiple sclerosis; a history of brain tumor, stroke, or other focal brain injury or head trauma; any acute medical condition, such as infections, nutritional deficiencies, or adverse drug reactions; a pacemaker or other implanted device; a history of kidney disease or diabetes; or claustrophobia. Forty‐five AHS2‐CAN study participants completed repeated cognitive assessment; 40 of those also completed the MRI scan. Of the five participants who did not complete the MRI, one had metal in his/her body, one was unable to lie flat due to back problems, and three declined due to symptoms of claustrophobia or concerns related to the contrast agent.

### 2.2 Cognitive assessment

During an in‐person visit, participants were administered a 2‐h neuropsychological battery, which included tests of verbal learning and memory [Rey Auditory Verbal Learning Test (RAVLT)(Rey, 1964), and Logical Memory subtest of the Wechsler Memory Scale–4th edition (Wechsler et al., 2008)], attention (Digit Span subtest of the Wechsler Adult Intelligence Scale–4th edition, WAIS‐IV (Wechsler et al., 2008)), processing speed (Coding subtest of the WAIS‐IV (Wechsler et al., 2008), Stroop Test (Golden et al., 1978), Trail‐Making Test (Reitan & Wolfson, 1993)), language (Boston Naming Test (Kaplan et al., 1983), FAS (Benton et al., 1989), Animals (Benton et al., 1989)), estimated premorbid verbal intelligence (American National Adult Reading Test, AMNART(Grober & Sliwinski, 1991)), and global cognitive functioning [Mini‐Mental State Examination (MMSE)(Folstein et al., 1975)] by trained psychometrists. The finger‐tapping test (FTT) (Strauss et al., 2006) was administered as a measure of fine motor control.

To reduce the number of variables in analyses, four composite cognitive scores were created to reflect domain‐specific abilities of memory (RAVLT Immediate Recall, RAVLT Delayed Recall, Logical Memory I and Logical Memory II); language (Boston Naming Test and Animals); processing speed (Trail‐Making Test Part A and Coding); and executive function (FAS, Trail‐Making Test Part B, and Digit Span). Individual test scores were first standardized by centering around the sample means and standard deviations; composite scores were then computed as averages of the individual component test scores. A fifth domain, motor control, was assessed with a single measure, the FTT, which was computed as the combined average score over five trials for both hands, with each trial representing the number of consecutive finger taps on a key completed within 10 s.

### 2.3 Neuroimaging

The MRI was collected on a 3T (Siemens Medical Systems, Erlangen, Germany) scanner using a 32‐channel array head coil. Total imaging time was 45 min and included 3D T1‐weighted MPRAGE with, TR = 1950 ms, TE = 2.3 ms, FA = 8 degrees, FOV = 240 × 240, matrix 256 × 256, acquisition time = 4:30, and slice thickness = 0.9 mm. A B1 corrected T1 map was created from four images with FA = 4,11,17,23, TR = 15, TE = 2.1 with the FOV and matrix the same as the DCE acquisition. A standard dose (0.2 ml/kg) of Multihance (gadobenate dimeglumine) was injected 45 s after the start of DCE sequence at 2 ml/s using a power injector. DCE was acquired in a coronal orientation with TR = 3.76 ms, TE = 1.35 ms, flip angle = 15°, slice thickness = 5 mm, FOV = 220 × 178 mm, matrix = 256 × 208, temporal resolution = 15.3 s, and total acquisition time = 12:44. FLAIR images were acquired with TR = 9000, TE = 81, TI = 2500, FOV = 220 × 213 mm, matrix = 320 × 217, and slice thickness = 4 mm with 1.2 mm gap.

### 2.4 Fatty acids

A drop of blood was collected on filter paper that was pre‐treated with an antioxidant cocktail (Fatty Acid Preservative Solution, FAPS) and allowed to dry at room temperature for 15 min. The dried blood spots (DBS) were shipped to OmegaQuant (Sioux Falls, SD) for fatty acid analysis as follows. One punch of the DBS was transferred to a screw‐cap glass vial followed by addition of BTM (methanol containing 14% boron trifluoride, toluene, methanol; 35:30:35 v/v/v) (Sigma‐Aldrich, St. Louis, MO). The vial was briefly vortexed and heated in a hot bath at 100˚C for 45 min. After cooling, hexane (EMD Chemicals, USA) and HPLC grade water were added, the tubes were recapped, vortexed, and centrifuged to help separate layers. An aliquot of the hexane layer was transferred to a GC vial. GC was carried out using a GC‐2010 Gas Chromatograph (Shimadzu Corporation, Columbia, MD) equipped with a SP‐2560, 100‐m fused silica capillary column (0.25 mm internal diameter, 0.2 μm film thickness; Supelco, Bellefonte, PA).

Fatty acids were identified by comparison with a standard mixture of fatty acids characteristic of red blood cells (RBC) (GLC OQ‐A; NuCheck Prep, Elysian, MN), which was also used to construct individual fatty acid calibration curves. Fatty acid composition was expressed as a percent of total identified fatty acids. In order to estimate fatty intake over a period of 120 days, the typical lifespan of erythrocytes (Arab, 2003; Tan et al., 2012), two RBC index scores were calculated by regressing component fatty acids with proprietary equations (OmegaQuant).

A long chain omega‐3 score reflected the combined amount of eicosapentaenoic acid (EPA; cis n‐3 20:5n) and docosahexaenoic acid (DHA; cis n‐3 22:6) in the erythrocytes. An omega‐6 score reflected arachidonic acid (AA; cis n‐6 20:4) in the erythrocytes. The correlation between the whole blood and RBC EPA and DHA levels was very strong (*r* > 0.95), while the AA correlation was moderate (*r* = 0.50).

### 2.5 Image processing

FLAIR images were used to determine the volume of white matter lesions. Lesions were segmented by the lesion prediction algorithm (Schmidt, 2017) as implemented in the LST toolbox version 3.0.0 (www.statistical‐modelling.de/lst.html) for SPM.

DCE data were processed with the software package ROCKETSHIP (Barnes et al., 2015). The dynamic DCE series was motion corrected using AFNI 3dvolreg. First, brain extraction was performed using the automated brain extration tool HD‐BET (Isensee et al., 2019). AFNI 3dvolreg was applied using a brain mask excluding the scalp which moves independently from the brain; heptic interpolation was utilized to minimize Gibbs ringing. An arterial input function (AIF) was selected from the jugular veins. The Patlak model, the most sensitive model for detecting subtle changes in the BBB (Barnes et al., 2015), was used to calculate *K*_trans_ values as measures of BBB integrity, with higher *K*_trans_ values reflecting worse BBB integrity. Regions of Interest (ROIs) were manually drawn in the hippocampus, superior corona radiata, corpus callosum, thalamus, caudate nucleus, and internal capsule, as these regions have been shown to have BBB changes associated with cognitive changes (Montagne et al., 2015). ROIs drawn in an area where the Patlak model failed to converge on a positive *K*_trans_ value for a given individual subject were excluded from analysis; this resulted in an exclusion of 31(13%) of 240 possible ROIs. Given the limited sample size and exploratory nature of this study, we did not examine the left and right sides of brain regions separately. For the hippocampus, both sides were drawn and then averaged together; for all other regions, the ROI was preferentially drawn on the right side, unless there were artifacts in the region, in which case the left was used. ROI definition was done blinded to any subject information about study participants and finalized before any statistical analyses.

The study was approved by the Loma Linda University Institutional Review Board, and written informed consent was obtained from all participants.

### 2.6 Statistics

Age‐ and sex‐adjusted Pearson correlation coefficients were calculated between fatty acid scores and the composite cognitive scores. Pearson correlation coefficients between fatty acid scores and *K*_trans_ values, and between *K*_trans_ values and composite cognitive scores were adjusted for age, sex, and white matter lesion (WML) volume. A two‐tailed *p*‐value < .05 was considered statistically significant. All analyses used SPSS 25 (IBM Corp., Armonk, N.Y., USA). Due to the small sample size and the exploratory nature of this study, we chose to limit the number to statistical comparisons as much as possible instead of performing multiple comparison corrections. As such, for fatty acids analysis, 24 variables were collected reflecting the levels of individual fatty acids, but only two composite fatty acids variables (omega‐3 score and omega‐6 score) were analyzed. For cognitive testing, 17 variables were collected, but five cognitive composite scores were analyzed (composite scores for memory, language, processing speed, executive function, and motor control). For DCE data, only six brain regions were analyzed (hippocampus, superior corona radiata, corpus callosum, thalamus, caudate nucleus, and internal capsule). The analyzed variables from the three primary outcomes were compared resulting in 52 statistical tests. For any statistically significant correlations (*p* < .05) between composites, we conducted post‐hoc analyses to examine individual correlations between the component variables; this resulted in 36 post hoc statistical comparisons. The data that support the findings of this study are available from the corresponding author upon reasonable request.

## 3 RESULTS

The mean age of participants (*n* = 45) was 76.8 years (SD = 8.6, min age = 63 years, max = 97 years). Participants were predominantly white, followed vegetarian diets, and were well educated. Their estimated VIQ (verbal IQ, M = 119; SD = 6.91) indicated high levels of premorbid intelligence (Table 1). There were no substantial differences in demographic factors between participants who participated in the MRI portion of the study (*n* = 40) and those who did not (Table 2).

**Table 1:** Characteristics of study participants

	Mean ± SD (range) or *n* (%)	
Characteristic	All participants(*n* = 45)	Participants with MRI data (*n* = 40)
Age, years	76.8 ± 8.6 (63–97)	76.3 ± 8.3 (63–90)
Sex		
Male	21 (46.7%)	18 (45.0%)
Female	24 (53.3%)	22 (55.0%)
Race		
White	39 (86.7%)	34 (85.0%)
Non‐white	6 (13.3%)	6 (15.0%)
Education, years	16.7 ± 2.5	16.9 ±2.4
MMSE	28.9 ± 1.5 (23–30)	28.9 ± 1.5 (23–30)
MOCA	25.5 ± 3.0 (19–30)	25.5 ± 3.0 (19–30)
GDS	2.5 ± 3.1 (0–14)	2.7 ± 3.2 (0–14)
AMNART VIQ	119.1 ± 6.9 (95–129)	119.5 ± 6.1 (105–129)
Vegetarian diet, yes*	25 (65.8)	22 (64.7)
BMI	25.7 ± 5.1	25.7 ± 5.3
Normal or underweight (BMI ≤ 24.9)	22 (49.9)	20 (50.0)
Overweight (BMI 25–29.9)	19 (42.2)	16 (40.0)
Obese (BMI > 30)	4 (8.9)	4 (10.0)
Fatty acid^†^		
Omega‐3 Index	5.3 ± 1.8 (2.88–11.79)	5.4 ± 1.9 (2.88–11.79)
Omega‐6 Index	15.0 ± 1.2 (12.91–17.74)	14.9 ± 1.1 (12.91–17.74)

Abbreviations: AMNART VIQ, American National Adult Reading Test verbal IQ; BMI, body mass index; MRI, magnetic resonance imaging; MMSE, Mini‐Mental State Examination.

* *n* = 38.

^†^ As percent of total fatty acid.

**Table 2:** Descriptive statistics of overall performance on cognitive assessment (mean) and measured *K*_trans_ values (median)

	Mean/median ± SD (range)	
Cognitive domain	All participants(*n* = 45)	Participants with MRI data (*n* = 40)
Memory	0.23 ± 0.83 (−1.32 to 1.76)	0.34 ± 0.79 (−1.32 to 1.76)
Language	0.16 ± 0.84 (−2.29 to 1.58)	0.27 ± 0.77 (−1.92 to 1.58)
Executive	0.12 ± 0.71(−1.50 to 1.71)^†^	0.18 ± 0.69 (−1.50 to 1.71)‡
Speed	0.01 ± 0.91 (−1.81 to 1.85)†	0.10 ± 0.92 (−1.81 to 1.85)‡
FTT	44.70 ± 7.87 (17.20–60.90)	44.93 ± 7.94 (17.20–60.90)
**DCE brain region**		
Hippocampus		0.5 ± 0.26 (0.09–1.2)
Superior corona radiata		0.26 ± 0.23 (0.02–0.92)
Corpus callosum		0.31 ± 0.21 (0.03–0.83)
Thalamus		0.31 ± 0.20 (0.03–0.95)
Caudate nucleus		0.31 ± 0.32 (0.01–1.14)
Internal capsule		0.28 ± 0.23 (0.006–0.83)

Abbreviations: DCE, dynamic contrast enhanced; FTT, finger‐tapping test; MRI, magnetic resonance imaging; SD, standard deviation.

^†^ *n* = 44.

^‡^ *n* = 39.

**FIGURE 1:**
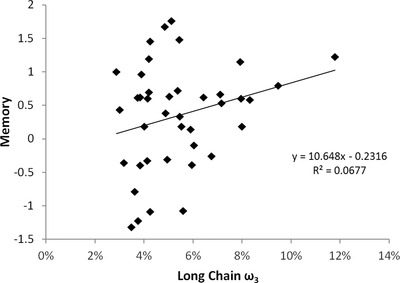
Correlation of participant's long chain omega‐3 values and cognitive memory scores, showing higher memory scores in participants with higher omega‐3 values. Pearson's correlation of 0.29 and 2‐tailed *p* = .06

Although we found no significant associations between omega‐3 scores and cognition, the long chain omega‐3 score was positively correlated with memory (Figure 1, *r *= 0.292; *p = *.058) and language (*r* = 0.267, *p* = .083), but did not reach statistical significance. Processing speed, executive function, and motor control did not show an association with omega‐3 (Table 3). The omega‐6 index was not statistically significantly correlated with any cognitive measures (Table 3).

**Table 3:** Age‐ and sex‐adjusted correlations (*p*‐value, 2‐tailed) between omega‐3 and omega‐6 scores and composite neuropsychological scores

	Adjusted with age/sex	Memory	Language	Executive	Speed	FTT
**Long chain** **omega‐3 score**	Pearson *r*	0.29	0.27	0.07	−0.02	0.19
	*p*	.06	.08	.68	.91	.22
**Omega‐6 score**	Pearson *r*	0.09	−0.09	−0.08	−0.08	–0.03
	*p*	.59	.58	.60	.60	.84

Number of subjects = 45 for memory, language, finger‐tapping test (FTT); 44 for executive, speed

**FIGURE 2:**
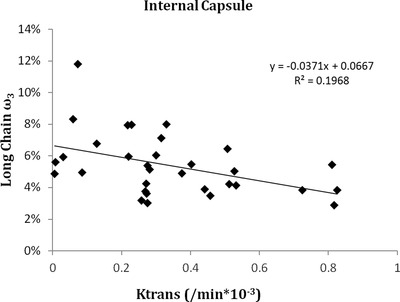
Correlation between the long chain omega‐3 values and the *K*_trans_ values in the internal capsule, showing significantly higher *K*_trans_ values (more BBB leakage) in participants with lower omega‐3 values. Pearson's correlation of −0.525 and 2‐tailed *p* = .004

The long chain omega‐3 score was negatively correlated with median *K*_trans_ values in the internal capsule (Figure 2, *r* = −0.3, *p* = .004). Correlations between the omega‐3 and the omega‐6 scores and other brain regions did not achieve statistical significance (Table 4). Post hoc analyses found that the correlation in the internal capsule was driven by a correlation with EPA (Table S1, *r* = −0.542, *p* = .003) and significantly correlated with DHA (*r* = −0.471, *p* = .01). *K*_trans_ values in the superior corona radiata were also significantly correlated with EPA (*r *= −0.372, *p* = .036).

**Table 4:** Correlations (*p*‐value, 2‐tailed) between brain regions *K*_trans_ and erythrocytes omega‐3 and omega‐6 scores adjusted for age, sex, and volume of white matter lesions

*K*_trans_ region	Adjusted with age/sex/WML	Long chain omega‐3 score	Omega‐6 score
Hippocampus	Pearson *r*	−0.113	0.065
	*p*	.53	.72
Superior corona radiata	Pearson *r*	−0.320	0.061
	*p*	.074	.74
Corpus callosum	Pearson *r*	−0.289	0.130
	*p*	.12	.50
Thalamus	Pearson *r*	−0.318	0.091
	*p*	.099	.65
Caudate nucleus	Pearson *r*	−0.127	0.046
	*p*	.48	.80
Internal capsule	Pearson *r*	−**0.525** **	0.076
	*p*	**.004**	.70

Abbreviation: WML, white matter lesion.

** bold *p* < 0.005

**FIGURE 3:**
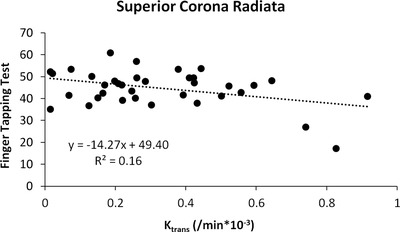
Scatterplot demonstrating inverse relationship between median *K*_trans_ values in the superior corona radiata and performance on the finger‐tapping test. Pearson's correlation of −0.426 and 2‐tailed *p* = .015

Median *K*_trans_ values in the superior corona radiata were statistically significantly correlated with FTT scores (Figure 3, *r *= −0.43, *p = *.015). Median *K*_trans_ values in the hippocampus, white matter, corpus callosum, thalamus, caudate nucleus, and internal capsule were not statistically significantly correlated with scores of memory, language, executive function, and speed (Table 5).

**Table 5:** Correlations between median *K*_trans_ values by region and neuropsychological tests, adjusted with age, sex, and volume of white matter lesions. Correlation is significant with *p* < .05, 2‐tailed

*K*_trans_ region	Adjusted with age/sex/WML	Memory	Language	Executive	Speed	FTT
Hippocampus	Pearson *r*	−0.026	−0.270	−0.189	−0.221	−0.104
	*p*	.89	.13	.29	.22	.57
Superior corona radiata	Pearson *r*	−0.115	−0.011	−0.031	0.180	−0.426*
	*p*	.53	.95	.87	.32	**.015**
Corpus callosum	Pearson *r*	−0.057	−0.151	−0.294	−0.151	‐0.233
	*P*	.77	.43	.12	.43	.22
Thalamus	Pearson *r*	−0.235	−0.274	−0.234	−0.119	−0.090
	*P*	.23	.16	.23	.55	.65
Caudate nucleus	Pearson *r*	−0.118	−0.234	−0.243	−0.274	−0.093
	*P*	.51	.19	.17	.12	.61
Internal capsule	Pearson *r*	−0.033	−0.007	−0.077	0.237	−0.334
	*P*	.87	.97	.70	.23	.083

Abbreviations: FTT, finger‐tapping test; WML, white matter lesion.

* bold *p* < 0.05

Of the ROIs we attempted to analyze, 13% were excluded as the *K*_trans_ values did not converge on a positive value. This may be due to excessive noise or motion artifacts in that area of the image. The *K*_trans_ values which did not converge were distributed across participants. However, since this is a substantial number of samples to exclude, we also performed all statistical analyses considering those regions as *K*_trans_ = 0. While this approach decreased the strength of the correlations, it did not change any of the statistically significant findings in our primary analysis or the conclusions of the study.

## 4 DISCUSSION

Omega‐3 PUFAs are essential components of cell membrane phospholipid bilayers and are also concentrated in synaptic membranes in the brain. They play an important role in brain physiology and biological activities, such as by increasing fluidity of neuronal membranes, modifying endothelial function and cerebral blood flow, synthesizing anti‐inflammatory mediators, and slowing degradation of nerve tissues (O' Donovan et al., 2019). High levels of omega‐3 PUFAs in the blood have also been associated with reduced cognitive decline in normal aging (Beydoun et al., 2007). Studies have shown a beneficial effect of omega‐3 PUFAs in neurological disorders due to anti‐inflammatory, anti‐apoptotic, and neuroprotective properties (Arab, 2003; Brookmeyer et al., 2007; Campbell et al., 2019; Harris & Thomas, 2010; Lin et al., 2012; Tan et al., 2012; Tu et al., 2013).

In our study, the long chain omega‐3 score was marginally associated with cognitive performance in the domains of memory and language, which are the most prominent areas of cognitive impairment associated with AD. Thus, our finding is consistent with previous literature which has demonstrated similar associations between omega‐3 levels, memory function, and risk of AD (Milte et al., 2011; Tan et al., 2012). Furthermore, higher omega‐3 levels have also been shown to reduce volume loss in the hippocampus, a region of the brain critical for learning and memory (Pottala et al., 2014). The relationship between omega‐3 PUFAs and other cognitive domains, including speed and executive function, is less clear, with some studies reporting a positive association (Milte et al., 2011; Tan et al., 2012), while others report no association (Ammann et al., 2013; Sydenham et al., 2012 ) or a negative association (Danthiir et al., 2014). Consistent with this literature, we did not observe associations with cognitive performance in the areas of processing speed or executive function.

Studies on animals have shown that omega‐3 PUFAs are important for preserving BBB integrity (Andreone et al., 2017). Our observation of an inverse correlation between long chain omega‐3 PUFAs and *K*_trans_ values in the internal capsule are thus in line with what would be expected from animal studies. To our knowledge, our study is the first to observe this in humans. The internal capsule is comprised of major ascending and descending white matter tracts, and significant changes in BBB integrity of the internal capsule have been measured in people with vascular risk factors and mild cognitive impairment (Montagne et al., 2020). At this time, it is not clear why associations were only detected in the internal capsule. It could be that the internal capsule is more protected by the omega‐3 PUFA, the BBB measurements are more sensitive in that area, or maybe associations in other brain regions were not detected due to insufficient statistical power. Our results provide evidence that the integrity of the BBB may be one of the mechanisms by which long chain omega‐3 PUFAs may be linked with brain health and protect from disease.

EPA is important in reducing neuroinflammation by competing against arachidonic acid for access to the same enzymes needed to produce pro‐inflammatory eicosanoids, such as prostaglandins, thromboxanes, leukotrienes, and other oxidized derivatives. Inflammatory mediators are known to disrupt the BBB leading to neurological dysfunction (Obermeier et al., 2013). In our study, correlations between long chain omega‐3 PUFAs and *K*_trans_ were driven by EPA, adding evidence of the potential importance of this omega‐3 PUFA in maintaining BBB integrity.

Finger tapping, which measures how quickly an individual can make repetitive taps on a key using their index finger, is a commonly used test of fine motor control of the upper extremities and requires functional integrity of the corticospinal tract, cerebellar motor circuitry, and proprioceptive pathways (Campbell et al., 2019). Age‐related declines in finger tapping frequency have been well‐documented (Bowden & McNulty, 2013; Rosjat et al., 2018). Our study found a strong correlation between finger tapping and *K*_trans_ in the superior corona radiata, a region that includes motor fibers of the corticospinal tract which help regulate fine motor functions of the upper extremities. A disruption in the BBB in corona radiata can affect fine motor functions, which also provides further evidence that disruption of the BBB is a key process in normal aging.

This was a small exploratory study and thus did not find statistically significant correlations between fatty acids and neuropsychological measures or between *K*_trans_ values, and some of the neuropsychological measures may have been a result of our being underpowered. Our cohort was exceptionally healthy with a relatively low rate of overall cognitive impairment and had a narrow range of variability in fatty acid values. In the general population, omega‐3 scores of ≤4% are considered high vascular risk, 4%–8% intermediate risk, and >8% is considered low risk (Harris & Von Schacky, 2004). Around 65% of the study cohort had omega‐3 scores putting them in an intermediate vascular risk group and only 7% was in the high‐risk group. Thus, a more heterogeneous sample with a greater number of participants with low omega‐3 scores would be useful for future studies. With cross‐sectional data, we cannot infer the directionality or temporality of the associations that we detected. It could be that declines in cognitive function precede BBB disruption, but we expect that reduction of BBB integrity as an underlying mechanism would be manifested through observable cognitive impairments (Huisa et al., 2015; Montagne et al., 2015). We adjusted correlations for age, sex, and white matter lesions, which does not rule out the potential that other factors such as education or comorbidities could confound the associations (Dias et al., 2014; Montagne et al., 2020). We also did not adjust correlations with omega‐3 and omega‐6 scores for other fatty acids to examine independent correlations. However, measurements of the individual fatty acids which contributed to these scores were expressed as a percentage of total fatty acids. Many of the variables we examined are not truly independent, so a Bonferroni correction for multiple testing was not done as this would be overly conservative, particularly given our limited sample size. Instead, we minimized the number of comparisons by reducing the number of variables examined in analyses using composite cognitive scores, omega‐3 and 6 scores, and a limited number of brain regions for *K*_trans_ values. A larger study which would allow more thorough analysis of individual cognitive tests, fatty acids, and a more diverse set of BBB measurements is merited.

In conclusion, we found significant correlations between long chain omega‐3 levels and BBB integrity and cognition, providing evidence of a possible mechanism by which omega‐3 may contribute to brain health.

## CONFLICT OF INTEREST

The authors declare no conflict of interest.

## Supporting information



Supporting Information

## References

[brb32273-bib-0001] Ammann, E. M., Pottala, J. V., Harris, W. S., Espeland, M. A., Wallace, R., Denburg, N. L., Carnahan, R. M., & Robinson, J. G. (2013). ω‐3 fatty acids and domain‐specific cognitive aging: Secondary analyses of data from WHISCA. Neurology, 81(17), 1484–1491. 10.1212/WNL.0b013e3182a9584c 24068783 PMC3888166

[brb32273-bib-0002] Andreone, B. J., Chow, B. W., Tata, A., Lacoste, B., Ben‐Zvi, A., Bullock, K., Deik, A. A., Ginty, D. D., Clish, C. B., & Gu, C. (2017). Blood‐brain barrier permeability is regulated by lipid transport‐dependent suppression of caveolae‐mediated transcytosis. Neuron, 94(3), 581–594.e5. 10.1016/j.neuron.2017.03.043 28416077 PMC5474951

[brb32273-bib-0003] Arab, L. (2003). Biomarkers of fat and fatty acid intake. Journal of Nutrition, 133(Suppl 3), 925S–932S. 10.1093/jn/133.3.925S 12612178

[brb32273-bib-0004] Barberger‐Gateau, P., Raffaitin, C., Letenneur, L., Berr, C., Tzourio, C., Dartigues, J. F., & Alpérovitch, A. (2007). Dietary patterns and risk of dementia: The Three‐City cohort study. Neurology, 69(20), 1921–1930. 10.1212/01.wnl.0000278116.37320.52 17998483

[brb32273-bib-0005] Barnes, S. R., Ng, T. S., Santa‐Maria, N., Montagne, A., Zlokovic, B. V., & Jacobs, R. E. (2015). ROCKETSHIP: A flexible and modular software tool for the planning, processing and analysis of dynamic MRI studies. BMC Medical Imaging, 15, 19. 10.1186/s12880-015-0062-3 26076957 PMC4466867

[brb32273-bib-0006] Benton, A., Hamsher, K., & Sivan, A. (1989). Multilingual aphasia examination. AJA Associates.

[brb32273-bib-0007] Beydoun, M. A., Kaufman, J. S., Satia, J. A., Rosamond, W., & Folsom, A. R. (2007). Plasma n‐3 fatty acids and the risk of cognitive decline in older adults: The Atherosclerosis Risk in Communities Study. American Journal of Clinical Nutrition, 85(4), 1103–1111. 10.1093/ajcn/85.4.1103 17413112

[brb32273-bib-0008] Bowden, J. L., & McNulty, P. A. (2013). The magnitude and rate of reduction in strength, dexterity and sensation in the human hand vary with ageing. Experimental Gerontology, 48(8), 756–765. 10.1016/j.exger.2013.03.011 23570975

[brb32273-bib-0009] Bowman, G. L., Silbert, L. C., Howieson, D., Dodge, H. H., Traber, M. G., Frei, B., Kaye, J. A., Shannon, J., & Quinn, J. F. (2012). Nutrient biomarker patterns, cognitive function, and MRI measures of brain aging. Neurology, 78(4), 241–249. 10.1212/WNL.0b013e3182436598 22205763 PMC3280054

[brb32273-bib-0010] Brookmeyer, R., Johnson, E., Ziegler‐Graham, K., & Arrighi, H. M. (2007). Forecasting the global burden of Alzheimer's disease. Alzheimer's Dement, 3(3), 186–191. 10.1016/j.jalz.2007.04.381 19595937

[brb32273-bib-0011] Butler, T. L., Fraser, G. E., Beeson, W. L., Knutsen, S. F., Herring, R. P., Chan, J., Sabaté, J., Montgomery, S., Haddad, E., Preston‐Martin, S., Bennett, H., & Jaceldo‐Siegl, K. (2008). Cohort profile: The Adventist Health Study‐2 (AHS‐2). International Journal of Epidemiology, 37(2), 260–265. 10.1093/ije/dym165 17726038

[brb32273-bib-0012] Campbell, W. W., Barohn, R. J., & DeJong, R. N. (2019). *DeJong's the neurologic examination*. Wolters Kluwer.

[brb32273-bib-0013] Conklin, S. M., Gianaros, P. J., Brown, S. M., Yao, J. K., Hariri, A. R., Manuck, S. B., & Muldoon, M. F. (2007). Long‐chain omega‐3 fatty acid intake is associated positively with corticolimbic gray matter volume in healthy adults. Neuroscience Letters, 421(3), 209–212. 10.1016/j.neulet.2007.04.086 17574755

[brb32273-bib-0014] Cramer, S. P., Modvig, S., Simonsen, H. J., Frederiksen, J. L., & Larsson, H. B. (2015). Permeability of the blood‐brain barrier predicts conversion from optic neuritis to multiple sclerosis. Brain, 138(Pt 9), 2571–2583. 10.1093/brain/awv203 26187333 PMC4547053

[brb32273-bib-0015] Danthiir, V., Hosking, D., Burns, N. R., Wilson, C., Nettelbeck, T., Calvaresi, E., Clifton, P., & Wittert, G. A. (2014). Cognitive performance in older adults is inversely associated with fish consumption but not erythrocyte membrane n‐3 fatty acids. Journal of Nutrition, 144(3), 311–320. 10.3945/jn.113.175695 24353345

[brb32273-bib-0016] Davidson, T. L., Monnot, A., Neal, A. U., Martin, A. A., Horton, J. J., & Zheng, W. (2012). The effects of a high‐energy diet on hippocampal‐dependent discrimination performance and blood‐brain barrier integrity differ for diet‐induced obese and diet‐resistant rats. Physiology & Behavior, 107(1), 26–33. 22634281 10.1016/j.physbeh.2012.05.015 PMC3409296

[brb32273-bib-0017] Dias, I. H., Polidori, M. C., & Griffiths, H. R. (2014). Hypercholesterolaemia‐induced oxidative stress at the blood‐brain barrier. Biochemical Society Transactions, 42(4), 1001–1005. 10.1042/BST20140164 25109993

[brb32273-bib-0018] Farrall, A. J., & Wardlaw, J. M. (2009). Blood‐brain barrier: Ageing and microvascular disease–systematic review and meta‐analysis. Neurobiology of Aging, 30(3), 337–352. 10.1016/j.neurobiolaging.2007.07.015 17869382

[brb32273-bib-0019] Folstein, M. F., Folstein, S. E., & McHugh, P. R. (1975). Mini‐mental state": A practical method for grading the cognitive state of patients for the clinician. Journal of Psychiatric Research, 12(3), 189–198. 10.1016/0022-3956(75)90026-6 1202204

[brb32273-bib-0020] Gatto, N. M., Garcia‐Cano, J., Irani, C., Liu, T., Arakaki, C., Fraser, G., Wang, C., & Lee, G. J. (2020). Observed physical function is associated with better cognition among elderly adults: The Adventist health study‐2. American Journal of Alzheimer's Disease and Other Dementias, 35, 153331752096086. 10.1177/1533317520960868 PMC10623938 32996324

[brb32273-bib-0021] Golden, C. J., Hammeke, T. A., & Purisch, A. D. (1978). Diagnostic validity of a standardized neuropsychological battery derived from Luria's neuropsychological tests. Journal of Consulting and Clinical Psychology, 46(6), 1258–1265. 10.1037/0022-006X.46.6.1258 730876

[brb32273-bib-0022] Grober, E., & Sliwinski, M. (1991). Development and validation of a model for estimating premorbid verbal intelligence in the elderly. Journal of Clinical and Experimental Neuropsychology, 13(6), 933–949. 10.1080/01688639108405109 1779032

[brb32273-bib-0023] Harris, W. S., & Thomas, R. M. (2010). Biological variability of blood omega‐3 biomarkers. Clinical Biochemistry, 43(3), 338–340. 10.1016/j.clinbiochem.2009.08.016 19733159

[brb32273-bib-0024] Harris, W. S., & Von Schacky, C. (2004). The Omega‐3 Index: A new risk factor for death from coronary heart disease? Preventive Medicine, 39(1), 212–220. 10.1016/j.ypmed.2004.02.030 15208005

[brb32273-bib-0025] Hooijmans, C. R., Pasker‐de Jong, P. C., de Vries, R. B., & Ritskes‐Hoitinga, M. (2012). The effects of long‐term omega‐3 fatty acid supplementation on cognition and Alzheimer's pathology in animal models of Alzheimer's disease: A systematic review and meta‐analysis. Journal of Alzheimer's Disease, 28(1), 191–209. 10.3233/JAD-2011-111217 22002791

[brb32273-bib-0026] Huisa, B. N., Caprihan, A., Thompson, J., Prestopnik, J., Qualls, C. R., & Rosenberg, G. A. (2015). Long‐term blood‐brain barrier permeability changes in Binswanger disease. Stroke; A Journal of Cerebral Circulation, 46(9), 2413–2418. 10.1161/STROKEAHA.115.009589 PMC4550546 26205374

[brb32273-bib-0027] Isensee, F., Schell, M., Pflueger, I., Brugnara, G., Bonekamp, D., Neuberger, U., Wick, A., Schlemmer, H.‐P., Heiland, S., Wick, W., Bendszus, M., Maier‐Hein, K. H., & Kickingereder, P. (2019). Automated brain extraction of multisequence MRI using artificial neural networks. Human Brain Mapping, 40(17), 4952–4964. 10.1002/hbm.24750 31403237 PMC6865732

[brb32273-bib-0028] Kalmijn, S., van Boxtel, M. P., Ocké, M., Verschuren, W. M., Kromhout, D., & Launer, L. J. (2004). Dietary intake of fatty acids and fish in relation to cognitive performance at middle age. Neurology, 62(2), 275–280. 10.1212/01.WNL.0000103860.75218.A5 14745067

[brb32273-bib-0029] Kanoski, S. E., Zhang, Y., Zheng, W., & Davidson, T. L. (2010). The effects of a high‐energy diet on hippocampal function and blood‐brain barrier integrity in the rat. Journal of Alzheimer's Disease, 21(1), 207–219. 10.3233/JAD-2010-091414 PMC4975946 20413889

[brb32273-bib-0030] Kaplan, E., Goodglass, H., Weintraub, S., & Goodglass, H. (1983). Boston naming test. Lea & Febiger.

[brb32273-bib-0031] Kuo, Y. T., So, P. W., Parkinson, J. R., Yu, W. S., Hankir, M., Herlihy, A. H., Goldstone, A. P., Frost, G. S., Wasserfall, C., & Bell, J. D. (2010). The combined effects on neuronal activation and blood‐brain barrier permeability of time and n‐3 polyunsaturated fatty acids in mice, as measured in vivo using MEMRI. Neuroimage, 50(4), 1384–1391. 10.1016/j.neuroimage.2010.01.057 20097292

[brb32273-bib-0032] Lin, P. Y., Chiu, C. C., Huang, S. Y., & Su, K. P. (2012). A meta‐analytic review of polyunsaturated fatty acid compositions in dementia. Journal of Clinical Psychiatry, 73(9), 1245–1254. 10.4088/JCP.11r07546 22938939

[brb32273-bib-0033] Milte, C. M., Sinn, N., Street, S. J., Buckley, J. D., Coates, A. M., & Howe, P. R. (2011). Erythrocyte polyunsaturated fatty acid status, memory, cognition and mood in older adults with mild cognitive impairment and healthy controls. Prostaglandins Leukotrienes and Essential Fatty Acids, 84(5–6), 153–161. 10.1016/j.plefa.2011.02.002 21392955

[brb32273-bib-0034] Montagne, A., Barnes, S. R., Sweeney, M. D., Halliday, M. R., Sagare, A. P., Zhao, Z., Toga, A. W., Jacobs, R. E., Liu, C. Y., Amezcua, L., Harrington, M. G., Chui, H. C., Law, M., & Zlokovic, B. V. (2015). Blood‐brain barrier breakdown in the aging human hippocampus. Neuron, 85(2), 296–302. 10.1016/j.neuron.2014.12.032 25611508 PMC4350773

[brb32273-bib-0035] Montagne, A., Nation, D. A., Sagare, A. P., Barisano, G., Sweeney, M. D., Chakhoyan, A., Pachicano, M., Joe, E., Nelson, A. R., D'Orazio, L. M., Buennagel, D. P., Harrington, M. G., Benzinger, T. L. S., Fagan, A. M., Ringman, J. M., Schneider, L. S., Morris, J. C., Reiman, E. M., Caselli, R. J., … & Zlokovic, B. V. (2020). APOE4 leads to blood‐brain barrier dysfunction predicting cognitive decline. Nature, 581(7806), 71–76. 10.1038/s41586-020-2247-3 32376954 PMC7250000

[brb32273-bib-0036] Morris, M. C., Evans, D. A., Bienias, J. L., Tangney, C. C., Bennett, D. A., Wilson, R. S., Aggarwal, N., & Schneider, J. (2003). Consumption of fish and n‐3 fatty acids and risk of incident Alzheimer disease. Archives of Neurology, 60(7), 940–946. 10.1001/archneur.60.7.940 12873849

[brb32273-bib-0037] Nation, D. A., Sweeney, M. D., Montagne, A., Sagare, A. P., D'Orazio, L. M., Pachicano, M., Sepehrband, F., Nelson, A. R., Buennagel, D. P., Harrington, M. G., Benzinger, T. L. S., Fagan, A. M., Ringman, J. M., Schneider, L. S., Morris, J. C., Chui, H. C., Law, M., Toga, A. W., & Zlokovic, B. V. (2019). Blood‐brain barrier breakdown is an early biomarker of human cognitive dysfunction. Nature Medicine, 25(2), 270–276. 10.1038/s41591-018-0297-y PMC6367058 30643288

[brb32273-bib-0038] O' Donovan, F., Carney, S., Kennedy, J., Hayes, H., Pender, N., Boland, F., & Stanton, A. (2019). Associations and effects of omega‐3 polyunsaturated fatty acids on cognitive function and mood in healthy adults: A protocol for a systematic review of observational and interventional studies. BMJ Open, 9(6), e027167. 10.1136/bmjopen-2018-027167 PMC6596976 31230010

[brb32273-bib-0039] Obermeier, B., Daneman, R., & Ransohoff, R. M. (2013). Development, maintenance and disruption of the blood‐brain barrier. Nature Medicine, 19(12), 1584–1596. 10.1038/nm.3407 PMC4080800 24309662

[brb32273-bib-0040] Pottala, J. V., Yaffe, K., Robinson, J. G., Espeland, M. A., Wallace, R., & Harris, W. S. (2014). Higher RBC EPA + DHA corresponds with larger total brain and hippocampal volumes: WHIMS‐MRI study. Neurology, 82(5), 435–442. 10.1212/WNL.0000000000000080 24453077 PMC3917688

[brb32273-bib-0041] Reitan, R. M., & Wolfson, D. (1993). The Halstead‐Reitan neuropsychological test battery theory and clinical interpretation. Neuropsychology Press.

[brb32273-bib-0042] Rey, A. (1964). L'examen clinique en psychologie. Presses universitaires de France.

[brb32273-bib-0043] Rizzo, N. S., Jaceldo‐Siegl, K., Sabate, J., & Fraser, G. E. (2013). Nutrient profiles of vegetarian and nonvegetarian dietary patterns. Journal of the Academy of Nutrition and Dietetics, 113(12), 1610–1619. 10.1016/j.jand.2013.06.349 23988511 PMC4081456

[brb32273-bib-0044] Rosjat, N., Liu, L., Wang, B. A., Popovych, S., Tóth, T., Viswanathan, S., Grefkes, C., Fink, G. R., & Daun, S. (2018). Aging‐associated changes of movement‐related functional connectivity in the human brain. Neuropsychologia, 117, 520–529. 10.1016/j.neuropsychologia.2018.07.006 30003904

[brb32273-bib-0045] Samieri, C., Maillard, P., Crivello, F., Proust‐Lima, C., Peuchant, E., Helmer, C., Amieva, H., Allard, M., Dartigues, J. ‐. F., Cunnane, S. C., Mazoyer, B. M., & Barberger‐Gateau, P. (2012). Plasma long‐chain omega‐3 fatty acids and atrophy of the medial temporal lobe. Neurology, 79(7), 642–650. 10.1212/WNL.0b013e318264e394 22855869

[brb32273-bib-0046] Schmidt, P. (2017). Bayesian inference for structured additive regression models for large‐scale problems with applications to medical imaging. Ludwig‐Maximilians‐Universität München, Germany. http://nbn‐resolving.de/urn:nbn:de:bvb:19‐203731

[brb32273-bib-0047] Sparks, D. L., Kuo, Y. M., Roher, A., Martin, T., & Lukas, R. J. (2000). Alterations of Alzheimer's disease in the cholesterol‐fed rabbit, including vascular inflammation. Preliminary observations. Annals of the New York Academy of Sciences, 903, 335–344. 10.1111/j.1749-6632.2000.tb06384.x 10818523

[brb32273-bib-0048] Starr, J. M., Farrall, A. J., Armitage, P., McGurn, B., & Wardlaw, J. (2009). Blood‐brain barrier permeability in Alzheimer's disease: A case‐control MRI study. Psychiatry Research, 171(3), 232–241. 10.1016/j.pscychresns.2008.04.003 19211227

[brb32273-bib-0049] Strauss, E., Sherman, E. M. S., & Spreen, O. (2006). A compendium of neuropsychological tests: Administration, norms, and commentary, 3rd ed. Oxford University Press.

[brb32273-bib-0050] Su, M. Y., Head, E., Brooks, W. M., Wang, Z., Muggenburg, B. A., Adam, G. E., Sutherland, R., Cotman, C. W., & Nalcioglu, O. (1998). Magnetic resonance imaging of anatomic and vascular characteristics in a canine model of human aging. Neurobiology of Aging, 19(5), 479–485. 10.1016/S0197-4580(98)00081-5 9880050

[brb32273-bib-0051] Sydenham, E., Dangour, A. D., & Lim, W. S. (2012). Omega 3 fatty acid for the prevention of cognitive decline and dementia. Cochrane Database of Systematic Reviews (Online), 13(6), Cd005379. 10.1002/14651858.CD005379.pub3 PMC11830470 22696350

[brb32273-bib-0052] Taheri, S., Gasparovic, C., Shah, N. J., & Rosenberg, G. A. (2011). Quantitative measurement of blood‐brain barrier permeability in human using dynamic contrast‐enhanced MRI with fast T1 mapping. Magnetic Resonance in Medicine, 65(4), 1036–1042. 10.1002/mrm.22686 21413067 PMC4950947

[brb32273-bib-0053] Takechi, R., Galloway, S., Pallebage‐Gamarallage, M. M., Lam, V., Dhaliwal, S. S., & Mamo, J. C. (2013). Probucol prevents blood‐brain barrier dysfunction in wild‐type mice induced by saturated fat or cholesterol feeding. Clinical and Experimental Pharmacology & Physiology, 40(1), 45–52. 23167559 10.1111/1440-1681.12032

[brb32273-bib-0054] Tan, Z. S., Harris, W. S., Beiser, A. S., Au, R., Himali, J. J., Debette, S., Pikula, A., DeCarli, C., Wolf, P. A., Vasan, R. S., Robins, S. J., & Seshadri, S. (2012). Red blood cell ω‐3 fatty acid levels and markers of accelerated brain aging. Neurology, 78(9), 658–664. 10.1212/WNL.0b013e318249f6a9 22371413 PMC3286229

[brb32273-bib-0055] Tantamango‐Bartley, Y., Jaceldo‐Siegl, K., Fan, J., & Fraser, G. (2013). Vegetarian diets and the incidence of cancer in a low‐risk population. Cancer Epidemiology, Biomarkers & Prevention, 22(2), 286–294. 10.1158/1055-9965.EPI-12-1060 PMC3565018 23169929

[brb32273-bib-0056] Titova, O. E., Sjögren, P., Brooks, S. J., Kullberg, J., Ax, E., Kilander, L., Riserus, U., Cederholm, T., Larsson, E.‐M., Johansson, L., Ahlström, H., Lind, L., Schiöth, H. B., & Benedict, C. (2013). Dietary intake of eicosapentaenoic and docosahexaenoic acids is linked to gray matter volume and cognitive function in elderly. Age (Dordr), 35(4), 1495–1505. 10.1007/s11357-012-9453-3 22791395 PMC3705118

[brb32273-bib-0057] Tu, W. C., Mühlhäusler, B. S., Yelland, L. N., & Gibson, R. A. (2013). Correlations between blood and tissue omega‐3 LCPUFA status following dietary ALA intervention in rats. Prostaglandins Leukotrienes and Essential Fatty Acids, 88(1), 53–60. 10.1016/j.plefa.2012.04.005 22521090

[brb32273-bib-0058] van Gelder, B. M., Tijhuis, M., Kalmijn, S., & Kromhout, D. (2007). Fish consumption, n‐3 fatty acids, and subsequent 5‐y cognitive decline in elderly men: The Zutphen Elderly Study. American Journal of Clinical Nutrition, 85(4), 1142–1147. 10.1093/ajcn/85.4.1142 17413117

[brb32273-bib-0059] Wang, H., Golob, E. J., & Su, M. Y. (2006). Vascular volume and blood‐brain barrier permeability measured by dynamic contrast enhanced MRI in hippocampus and cerebellum of patients with MCI and normal controls. Journal of Magnetic Resonance Imaging, 24(3), 695–700. 10.1002/jmri.20669 16878309

[brb32273-bib-0060] Wechsler, D., Psychological, C., & Pearson Education, I. (2008). WAIS‐IV: Wechsler adult intelligence scale.

[brb32273-bib-0061] Zlokovic, B. V. (2011). Neurovascular pathways to neurodegeneration in Alzheimer's disease and other disorders. Nature Reviews Neuroscience, 12(12), 723–738. 10.1038/nrn3114 22048062 PMC4036520

