# Supplementary material for: Omega‐3 fatty acids are associated with blood–brain barrier integrity in a healthy aging population
Source: Brain Behav. 2021 Jul 29;11(8):e2273. doi: 10.1002/brb3.2273 (PMC8413753; doi:10.1002/brb3.2273)
Supplement: Supplementary file 1 — Supporting Information [file BRB3-11-e2273-s001.docx]

**SUPPLEMENTAL TABLES**

| **K_trans_ Region** | **Adjusted for Age/Sex/WML** | **ALA** | **EPA** | **DHA** | **LA** | **GLA** | **AA** |
| --- | --- | --- | --- | --- | --- | --- | --- |
| Hippocampus | Pearson *r* | -0.030 | -0.190 | -0.054 | 0.019 | 0.047 | 0.065 |
|  | *p* | 0.87 | 0.29 | 0.77 | 0.92 | 0.80 | 0.71 |
| Superior Corona Radiata | Pearson *r* | 0.231 | **-0.372*** | -0.262 | -0.023 | 0.066 | 0.061 |
|  | *p* | 0.20 | **0.036** | 0.15 | 0.90 | 0.72 | 0.74 |
| Corpus Callosum | Pearson *r* | 0.222 | -0.285 | -0.270 | -0.236 | -0.040 | 0.130 |
|  | *p* | 0.24 | 0.13 | 0.15 | 0.21 | 0.84 | 0.50 |
| Thalamus | Pearson *r* | 0.072 | -0.344 | -0.278 | 0.024 | 0.076 | 0.091 |
|  | *p* | 0.72 | 0.07 | 0.15 | 0.91 | 0.70 | 0.646 |
| Caudate Nucleus | Pearson *r* | 0.049 | -0.202 | -0.069 | -0.113 | 0.104 | 0.046 |
|  | *p* | 0.80 | 0.26 | 0.71 | 0.53 | 0.56 | 0.798 |
| Internal Capsule | Pearson *r* | -0.169 | **-0.542*** | **-0.471*** | 0.043 | -0.285 | 0.076 |
|  | *p* | 0.39 | **0.003** | **0.011** | 0.83 | 0.14 | 0.70 |

**Supplemental Table 1:** Correlation between brain regions K_trans_ and whole blood fatty acids (ALA, EPA, DHA, LA, GLA and AA), adjusted for age, sex, and volume of white matter lesions. Correlation is significant with p<0.05, in bold (2-tailed). ALA=Alpha-Linolenic Acid; EPA=Eicosapentaenoic acid; DHA=Docosahexaenoic Acid; LA=Linoleic Acid; GLA=Gamma-Linolenic Acid; AA=Arachidonic Acid; WML=white matter lesions.
